# Insights into Sexism: Male Status and Performance Moderates Female-Directed Hostile and Amicable Behaviour

**DOI:** 10.1371/journal.pone.0131613

**Published:** 2015-07-15

**Authors:** Michael M. Kasumovic, Jeffrey H. Kuznekoff

**Affiliations:** 1 Ecology and Evolution Research Centre, The University of New South Wales, Sydney, NSW, 2052, Australia; 2 Department of Integrative Studies, Miami University Middletown, 4200 N. University Blvd., Middletown, OH, 45042-3497, United States of America; Universidad de Alicante, ITALY

## Abstract

Gender inequality and sexist behaviour is prevalent in almost all workplaces and rampant in online environments. Although there is much research dedicated to understanding sexist behaviour, we have almost no insight into what triggers this behaviour and the individuals that initiate it. Although social constructionist theory argues that sexism is a response towards women entering a male dominated arena, this perspective doesn’t explain why only a subset of males behave in this way. We argue that a clearer understanding of sexist behaviour can be gained through an evolutionary perspective that considers evolved differences in intra-sexual competition. We hypothesised that female-initiated disruption of a male hierarchy incites hostile behaviour from poor performing males who stand to lose the most status. To test this hypothesis, we used an online first-person shooter video game that removes signals of dominance but provides information on gender, individual performance, and skill. We show that lower-skilled players were more hostile towards a female-voiced teammate, especially when performing poorly. In contrast, lower-skilled players behaved submissively towards a male-voiced player in the identical scenario. This difference in gender-directed behaviour became more extreme with poorer focal-player performance. We suggest that low-status males increase female-directed hostility to minimize the loss of status as a consequence of hierarchical reconfiguration resulting from the entrance of a woman into the competitive arena. Higher-skilled players, in contrast, were more positive towards a female relative to a male teammate. As higher-skilled players have less to fear from hierarchical reorganization, we argue that these males behave more positively in an attempt to support and garner a female player’s attention. Our results provide the clearest picture of inter-sexual competition to date, highlighting the importance of considering an evolutionary perspective when exploring the factors that affect male hostility towards women.

## Introduction

Gender equality is currently one of the most hotly discussed topics across all scientific disciplines, political and religious ideologies, and workplaces [1–3]. Although many scientific discussions explore how we can overcome gender inequalities [3,4], they highlight how little we know about the factors that trigger sexist behaviour and the individuals that instigate it. Understanding these factors is absolutely necessary to attaining equality. Although many sociologists and feminists argue that sexism can only be explored through social constructionist theory [5,6], such a view assumes that sexist behaviour is solely determined by social and cultural environments, ignoring biological variation. From the same perspective, a biological approach alone ignores the importance of social interactions. We argue that a much clearer understanding of sexist behaviour is reached using evolutionary theory as it incorporates both a social and biological perspective [7].

From an evolutionary perspective, intrasexual competition is common and is one of the strongest forces shaping sex differences [8]. This is also true in humans and the fact that direct conflicts were largely fought by men [9] can help explain sex differences in body structure [10,11]. In a similar manner, these biological sex differences also shaped sex-specific competitive strategies. As a man’s access to resources and mates (i.e. fitness) is determined by his position in a hierarchy [12–14], it is important for men to understand and navigate dominance hierarchies enforced through overt signals of dominance [15]. In contrast, there is no evidence that a woman’s fitness is determined by her position in a hierarchy, making overt hierarchical navigation less important. In addition, because direct conflicts are relatively more costly for women [16], women generally use more subtle competitive strategies that reduce the social status of rivals and minimize retaliation [17,18]. The sexes thus differ in how social hierarchies are navigated, and the correlation between the placement within a hierarchy and fitness.

Despite these historical sex-differences in competition, men and women currently interact and compete for the same professional positions and accolades [2,3]. The modern competitive environment has shifted in such a way that individual status is determined by performance metrics largely unrelated to physical differences. Nonetheless, gender differences in perceptions of hierarchies and behaviours associated with negotiating them could potentially explain the increased hostility women experience in competitive scenarios. Nowhere is this more apparent than in online environments where physical differences are inconsequential, yet women receive more than four-times the negative comments from men [19,20].

Video games perfectly blend biologically and socially-constructed competitive environments thus providing a unique opportunity to probe how intersexual competition shapes individual behaviours and attitudes in humans. For example, a recent survey by the Entertainment Software Association states men and women are equally likely to play competitive video games [21]. Because these aggressive virtual competitions can evoke meaningful neural [22,23], physiological [24,25], and behavioural [26,27] outcomes, these outcomes may parallel those seen in actual physical competitions, although the competitions do not require physical superiority since outcomes are determined by response times and cognitive capacity rather than physical strength. Nonetheless, video games remain a bastion of sexual stereotypes and inequality for several reasons. First, men are often graphically depicted as aggressive and hypermasculine, while women are portrayed in an overly sexualized manner [28,29] and are more often depicted as damsels in distress [30]. Second, anecdotal evidence suggests that men play more online competitive games than women (or at least that women may avoid speaking while playing) suggesting that some games may be considered ‘boys games’. The sexualized environment combined with men being the overwhelming vocal majority suggests an environment not unlike many current work environments where women can represent as little as 10% of the professional work force (e.g., electrical engineering, [31]). This suggests that competitive online video games may represent a common phenomenon that women encounter. Additionally, it reinforces the fact that women are entering a male-dominated competitive arena where they must compete in a direct fashion along an overt dominance hierarchy, rather than the more indirect route accustomed to through evolutionary history.

To explore social constructionist and evolutionary explanations for sexist behaviour, we examine how individual performance and social standing affect female-directed male behaviour using an online first-person shooter video game, *Halo 3* (see methods for greater details regarding the game). According to social constructionist theory, men behave in a sexist manner towards women to remove them from a male-dominated arena (i.e. backlash) [6]. Social constructionist theory thus predicts that focal male players should be relatively more negative and less positive towards a female (outgroup member) compared to a male-voiced teammate when encountered regardless of the focal player’s in-game performance or status.

In contrast, evolutionary theory suggests that sexist behaviour is in response to a threat to a male’s position in the hierarchy, which if reduced, limits his access to potential mates. Evolutionary theory thus predicts that a male’s behaviour should be moderated by status and performance, such that only lower-status males that have the most to lose with a hierarchical reorganization by the introduction of a female competitor will be hostile towards female players. It also predicts that higher-status males should decrease the frequency of negative comments and increase their frequency of positive comments as female-voiced players represent a potential mate. Evolutionary theory also makes a secondary prediction regarding male-male interactions: the frequency of positive and negative comments should follow a typical hierarchical response such that poorer performance and lower status should evoke submissive behaviour (more positive and fewer negative statements).

## Materials and Methods

### Ethics Statement

Neither written nor verbal consent to participate in this study was collected by the authors specifically because all individuals that participated in the study were anonymous (as players use pseudonyms) and had already agreed to the terms of Xbox Live (which state that conversations can be recorded). Because of these aspects, and that data for this study was obtained from a prior study, the Ohio University Institutional Review Board (IRB) determined that this study was exempt from IRB review under Category 4, research involving collection or study of existing data.

### Video Game Used

In multiplayer first-person shooters like *Halo 3*, teams cooperate to kill members of the opposing team outside of a dedicated storyline such as that seen in a single player campaign within the game. As a result, the multiplayer games can be argued to be direct competitions outside of any sexualized storyline or content as they are only associated with the goal of eliminating opponents. Killing opponents results in a positive outcome for the team, while dying results in a negative outcome. In addition, improved performance within a game allows players to attain higher ranks and improve their skill rating, a long-term measurement of player status (i.e. dominance) that is publicly exhibited. We examine how individuals behave toward a male or female-voiced teammate as a function of positive (number of kills) and negative (number of deaths) player performance, as well as player status (maximum skill achieved).

A secondary benefit of using *Halo 3* is that players are covered head-to-toe with armor and identified by armor color, rather than facial features or body type. Additionally, player controlled avatars are not the hypersexualized males normally seen in many other video games, hopefully decreasing the overt sexism seen in most online competitive games and minimizing the effect of the game environment.

### Data Collection

Data collection involved analyzing video recordings generated from Kuznekoff and Rose’s (2013) original study. In that study, the authors played in and recorded actual multiplayer matches of the video game *Halo 3*. The audio/video output of an Xbox 360 console was routed to a television and to a digital video converter connected to a computer. The converter allowed each multiplayer match to be recorded as a video file to the computer, capturing exactly what the researcher experienced while in the game. In addition, the researchers connected an audio playback device to the Xbox controller, which allowed them to broadcast pre-recorded audio clips to other players, as if they were speaking to each other through the real-time voice channel.

Three Xbox LIVE (XBL) accounts were created and assigned to a respective experimental manipulation: control, male, and female. The control condition simply played in matches of *Halo 3* as one normally would; however, this condition did not use the real-time voice channel to communicate with other players, and as a result, was not further analyzed here. For the male and female manipulations, the researchers also played in the matches as expected, but also developed a list of roughly a dozen phrases that were recorded separately by a male and female voice (S1 Text). These phrases were broadcast, during the games, to other players using the real-time voice channel. These prerecorded phrases were identical in the male and female condition, harmless in nature, and designed to be inoffensive. Phrases included: ‘I like this map’, ‘nice shot there’, ‘I had fun playing that game’, ‘I think I just saw a couple of them heading this way’, and ‘that was a good game everyone’.

In the *Halo 3* XBL matchmaking system, up to 8 individuals (4 on each team) are matched up against other players, by the system, according to their skill level. While players may form a party of up to four people beforehand, players have no control over the other players assigned to that game by the matchmaking system. The player skill level is an objective indicator (determined by an undisclosed algorithm by the developer) of how good or bad that particular player is in the specific playlist selected. This skill level ranges from 1–50, with a higher score indicating a greater skill level. While being matched, players can leave the game up until the game starts and any vacancies may be filled with other players before starting. During the matches, we broadcasted the pre-recorded audio clips to other players at appropriate times (S1 Text). For example, before the game would actually start, it would be appropriate to say ‘I like this map’; however, using that statement at the end of the game would make little sense and perhaps confuse other players. In every case, the audio clips could primarily be heard by teammates, and on occasion by opponents (if they were close enough to the experimental player). All the players on both teams could communicate with one another in the post-game lobby. Each multiplayer match was recorded as a separate video file and generally included not only what the player saw, but also what they said (i.e., pre-recorded audio statements) and how other players reacted.

### Coding

Following the original data collection, and using the suggestions for future research identified by Kuznekoff and Rose (2013) as a starting point, the current study focused on individual gamers instead of each overall game. First, we only used the recordings from the male and female manipulations in which other players spoke. Following this selection phase, the selected video files (*N* = 126) were then transcribed by a professional transcription company (www.pacifictranslation.com.au) by individuals blind to the true purpose of the study and the experimental manipulations used. The authors randomly spot-checked 10% of the files for accuracy. One independent coder and one of the authors coded the comments each player made. Coders were provided with a codebook that explained the coding procedures. Both coders looked for comments and questions that appeared to be directed toward the experimental player and classified them as positive, negative, or neutral. Comments and questions in the positive categories were those that coders perceived as supportive or friendly, while those in the negative categories were those that the coders perceived to be aggressive or condescending (S2 Text). Comments and questions were considered neutral if they did not fall in one of the previous categories. For analyses, we collapsed questions and comments of each type (negative, positive and neutral) to increase our power. We also explored whether the negative statements in the female manipulation could be considered hostile sexism [32].

In order to calculate intercoder reliability (ICR) statistics, 10% of the total transcript files (N = 263), one file for each individual player/participant, were randomly selected and coded by both coders. Cohen’s kappa was calculated for each category and kappa values between 0.61 and 0.80 represent substantial agreement [33]. The kappa values for each coding category in this study are as follows: 0.85 for negative comments, 0.72 for positive comments, 0.99 for negative questions, 0.88 for positive questions, and 0.93 for neutral statements.

### Game and Player Data

In addition to coding comments made by focal players, we also examined focal-player performance for the games they played in. These data included the overall game outcome (i.e., win or loss) from the perspective of the focal player, the focal player’s maximum skill level achieved, and the focal player’s number of kills and deaths. Lastly, these performance statistics were also recorded for the experimentally controlled player, which allowed us to calculate a relative performance metric for each focal player (focal player value—experimental player value). The skill level is calculated automatically by the *Halo 3* matchmaking system, which analyzes the performance of the player in each game and adjusts that player’s skill level over time. This skill level provides a standard metric by which player skill (i.e. dominance) can be compared against each other.

### Statistical analyses

We examined whether the number of positive, negative and neutral statements by a player were correlated with the outcome of the game, the experimental manipulation, and individual performance metrics (number of kills, deaths, and the maximum skill level achieved). We also added interactions between manipulations and the three performance metrics as we hypothesized that performance would differentially affect behaviour of focal individuals in the different manipulations.

Rather than individual performance alone, we also examined whether positive, negative and neutral statements were correlated with performance of the focal player relative to the experimental player (i.e. the experimenter controlled player). For this examination, we created a model with the experimental manipulation and each of the relative performance metrics. We also added the interactions between the manipulation and each of the relative performance metrics.

We used separate GLMs with a Poisson distribution and a log link to test the six models regarding positive, negative, and neutral statements outlined above. To examine whether individual performance relative to the experimental player was correlated with the use of sexist statements, we used a GLM with a binomial distribution and a logit link. All statistics were completed in R 3.1.1, mean values are followed with the standard error, and we provide standardized beta coefficients for significant effects. We used the effects package [34] to plot the predicted responses in all our figures. Data are available as supplementary information (S1 Dataset) and the data and R script are on Github (https://github.com/latrodektus/VG_Sexism).

## Results

We played a total of 163 games of Halo 3 in the two manipulations. We stopped at 163 as this is a substantial time effort. Data could not be analyzed during the experiment as the transcripts needed to be transcribed. Of the 163 games, 82 were in the female manipulation and 81were in the male manipulation and players only spoke within 102 of the games. A total of 189 players spoke in these 102 games; all of them were male. This is not to say that women did not play, just that they did not speak. This does, however, reinforce the fact that women are entering a very male dominated environment. Of these individuals, 147 individuals were teammates of the experimental player and 42 individuals were opponents. For our analyses, we focused on teammates as these individuals interact with the experimental player for the duration of the game. Opponents, in contrast, only interacted with the experimental player for a short period (less than 3 minutes) in the lobby after the game. Of the 147 teammates, 82 individuals were in the female manipulation and 65 were in the male manipulation. The range of players that spoke within an individual game varied from 1–3. To test whether the ‘Game’ affected the statement type and number, we added game as a random variable in our initial analyses. As ‘Game’ did not explain any of the variation in the type and number of statements made, it was removed from further analyses.

**Fig 1:**
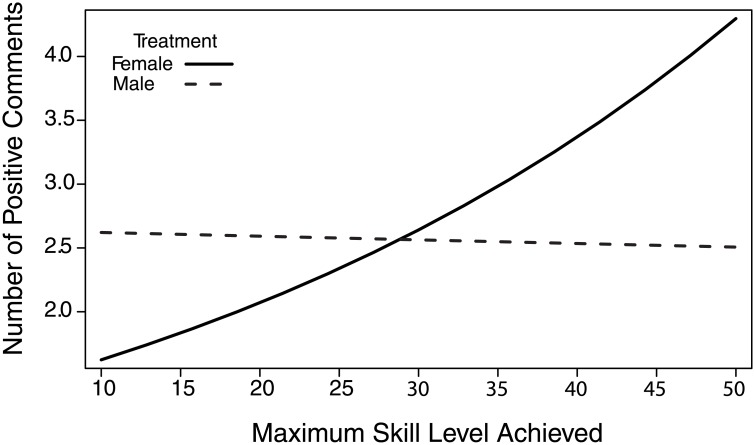
The predicted number of positive comments as a function of manipulation and maximum skill level achieved. The number of positive statements uttered by focal players increased in the female manipulation as the maximum skill level achieved by the focal player increased. There was no relationship between the number of positive comments uttered and the focal player’s maximum skill level achieved in the male manipulation.

We first examined whether game outcome, experimental manipulation, or metrics of individual performance affected the number of positive statements. There was a significant positive correlation between the number of positive statements and deaths (β = 0.20, χ^2^ = 14.61, p<0.0001, Table 1) with focal individuals that died more often stating more positive comments. There was also a significant interaction between experimental manipulation and the maximum skill level achieved by focal players (β = -0.31, χ^2^ = 7.01, p = 0.008) on the number of positive statements (Fig 1). In the female-voiced manipulation, lower-skilled players were less positive, while higher skilled players were more positive (Fig 1).

**Table 1:** Results of differences in performance.

	Positive statements		Negative statements		Neutral statements	
	χ^2^	P	χ^2^	P	χ^2^	P
Game Outcome	0.015	0.90	1.97	0.16	0.05	0.82
Experimental manipulation	0.87	0.35	5.88	**0.015**	0.46	0.50
Kills	0.0004	0.98	16.31	**<0.0001**	2.40	0.12
Deaths	14.61	**0.0001**	18.44	**<0.0001**	5.71	**0.017**
Max Skill Level	5.53	**0.018**	3.99	**0.046**	0.43	0.51
Manipulation × Kills	0.78	0.38	6.07	**0.014**	0.53	0.47
Manipulation × Deaths	0.005	0.94	5.44	**0.02**	0.01	0.92
Manipulation × Skill	7.01	**0.008**	0.412	0.52	2.42	0.12

The correlation between the number of positive, negative, and neutral statements as a function of game outcome, the experimental manipulation and the individual performance metrics.

**Fig 2:**
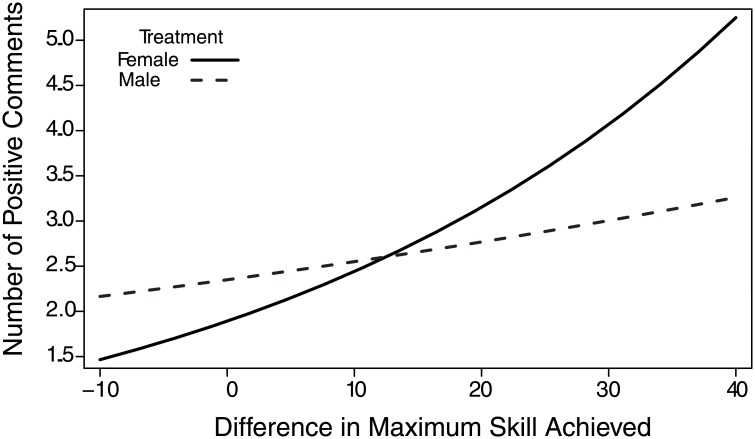
The predicted number of positive comments as a function of manipulation and the relative difference in maximum skill level achieved. The number of positive statements made by the focal player was positively correlated with the difference in the maximum skill achieved of the focal player relative to the experimental player, and was significantly affected by the experimental manipulation. A focal player with was more positive towards a female-voiced player when they had a greater relative difference in maximum skill level, and more negative towards a female-voiced teammate when they had a lower relative difference in maximum skill level.

We next examined whether the number of positive statements made by focal players was correlated with their performance metrics relative to the experimental player. There was a positive correlation between the number of positive statements and the relative difference in deaths (β = 0.13, χ^2^ = 9.44, p = 0.002) such that focal players that had a relatively greater number of deaths than the experimental player made more positive statements. Focal players with a higher skill achieved relative to the experimental player also made more positive comments (β = 0.29, χ^2^ = 5.12, p = 0.02); there was a near-significant interaction with manipulation such that the experimental player in the female-voiced treatment received more positive comments when focal players were of a higher skill level, but fewer positive comments when the experimental player was higher in skill (Fig 2). There was also a negative correlation between the relative difference in the number of kills and the number of positive statements (β = -0.17, χ^2^ = 9.05, p = 0.003) such that focal players that had relatively fewer kills than the experimental player made more positive statements. There was no effect of the manipulation or any of the other manipulation by performance interactions (Table 2).

**Table 2:** Results of relative-differences in performance.

	Positive statements		Negative statements		Neutral statements	
	χ^2^	P	χ^2^	P	χ^2^	P
Experimental manipulation	0.88	0.35	7.06	**0.008**	0.38	0.54
Skill Difference	5.12	**0.02**	0.18	0.67	1.51	0.22
Kill Difference	9.05	**0.003**	5.52	**0.019**	7.30	**0.007**
Death Difference	9.44	**0.002**	9.43	**0.002**	0.67	0.41
Manipulation × Skill	3.55	0.059	0.50	0.48	0.54	0.46
Manipulation × Kill	0.0006	0.98	1.46	0.23	0.004	0.94
Manipulation × Death	0.67	0.41	1.04	0.31	1.52	0.22

The correlation between the number of positive, negative, and neutral statements as a function of the experimental manipulation and the relative performance of the focal individuals compared to the experimental individual.

**Fig 3:**
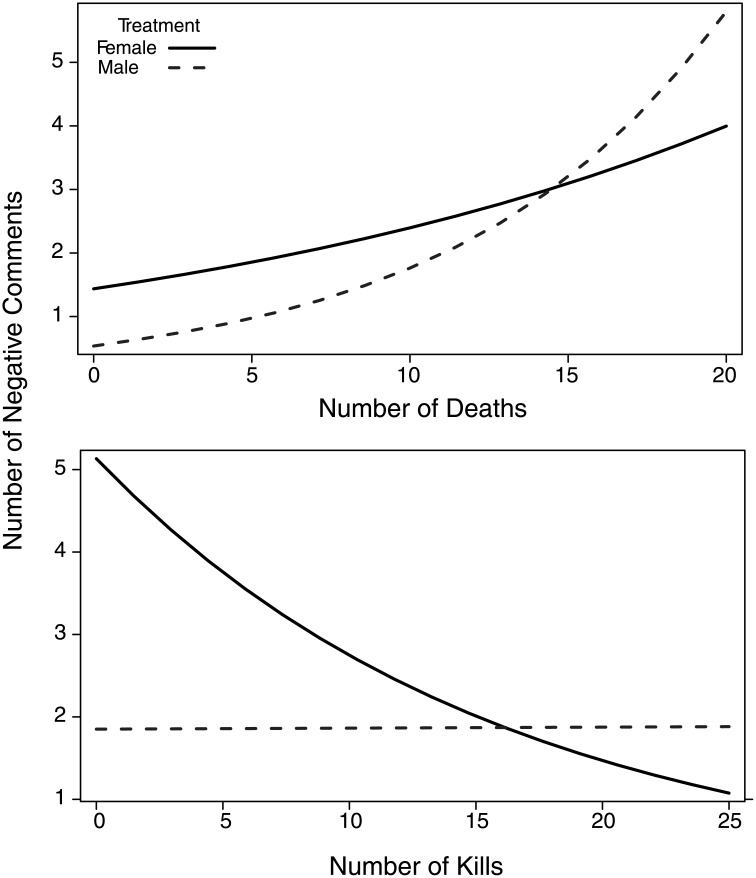
The predicted number of negative comments as a function of manipulation and the number of deaths (top) and kills (bottom). The number of negative statements uttered by the focal player was positively correlated with the number of times they died (top) with female-voiced teammates generally receiving more negative statements until approximately 15 deaths, after which male-voiced teammate received more negative statements. In contrast, focal players made more negative statements towards female-voiced teammates and decreased the number of negative comments the better they performed (i.e. with a greater number of kills; bottom).

For the examination of negative statements, there were two focal players in the female-voiced manipulation that made 10 more negative statements than the next highest individuals (greater than 5 standard deviations from the mean). As a result, we removed them from our analysis to ensure they did not skew our results towards significance. Focal players stated more negative statements in the female-voiced (2.66±0.36) compared to the male-voiced manipulation (1.90±0.34, χ^2^ = 5.88, p = 0.0015; Table 1). The maximum skill level achieved by focal players was negatively correlated with the number of negative statements (β = -0.18, χ^2^ = 3.99, p = 0.046). There was also a significant interaction of the manipulation with both measures of focal player performance. Overall, there were a greater number of negative comments made by focal players in both manipulations as they died more often (i.e. their team performance decreased). However, focal players made a greater number of negative statements in the female-voiced manipulation when dying less (i.e. greater performance), and a greater number of negative statements in the male-voiced manipulation when dying extremely often (i.e. very poor performance) (β = 0.28, χ^2^ = 5.44, p = 0.02; Fig 3). In contrast, there was no effect of individual performance (i.e. the number of kills) on the number of negative statements made by focal players in the male-voiced manipulation, but focal players in the female-voiced manipulation stated significantly more negative statements when their performance was poor (i.e. fewer kills) and stated fewer negative statements when performing exceptionally (β = 0.35, χ^2^ = 6.07, p = 0.014; Fig 3).

We next examined whether the number of negative statements made by focal players was correlated with their performance metrics relative to the experimental player. Again, there was also a greater number of negative statements in the female compared to the male experimental manipulation (χ^2^ = 7.06, p = 0.008). There was a positive correlation between the difference in deaths and the number of negative statements (β = 0.24, χ^2^ = 9.43, p = 0.002) with focal players that died relatively more often than the experimental player (i.e. relatively poorer team performance) making more negative comments. There was also a negative correlation between the relative difference in kills and the number of negative statements made (β = -0.16, χ^2^ = 5.52, p = 0.019) such that focal players that had relatively more kills than the experimental player made fewer negative comments. There was no effect of any other factors on the number of negative statements (Table 2).

The number of neutral comments stated by focal players was only affected by the number of deaths. Focal players that died more stated more neutral statements (β = 0.16, χ^2^ = 5.71, p = 0.017). In the model examining how relative performance and skill affected neutral statements, there was only a negative correlation between the number of neutral statements made by focal players and the difference in kills between the focal and experimental player (β = -0.17, χ^2^ = 7.30, p = 0.007) such that players that had a greater number of kills relative to the experimental player made fewer neutral comments. No other factors affected the number of neutral statements made by focal players.

Since there were a greater number of negative statements within the female manipulation, we examined whether these statements could be considered hostile sexism [32]. Of the 82 players in the female manipulation playing on the same team as the experimental player, only 11 individuals (13%) uttered hostile sexist statements. As a result of this small sample size, we only examined whether the presence of hostile sexist statements was affected by individual performance relative to the experimental player. We found that the presence of sexist statements was not determined by differences in maximum skill achieved (χ^2^ = 1.70, p = 0.19), the number of deaths (χ^2^ = 0.57, p = 0.45) or the number of kills (χ^2^ = 2.25, p = 0.13) relative to the experimental player.

## Discussion

The goal of this study was to examine the moderating effect of performance and skill on the frequency of positive and negative statements towards a female- or male-voiced teammate in an online first-person shooter video game—a metric providing insight into sexism. We found that skill determined the frequency of positive and negative statements spoken towards both male- and female-voiced teammates. In addition, poorer performance (fewer kills and more deaths) resulted in more negative statements specifically in the female-voiced manipulation. We thus argue that our results best support an evolutionary explanation of female-directed aggression. Low-status males that have the most to lose due to a hierarchical reconfiguration are responding to the threat female competitors pose. High-status males with the least to fear were more positive, suggesting they were switching to a supportive, and potentially, mate attraction role.

Our results are particularly important because the ecological design observed players in their natural state rather than in a laboratory or other foreign environment. The large online community of players using pseudonyms means that policing individual behaviour is almost impossible. Individuals thus generally behave expressively and honestly since there is no fear of retribution. Additionally, our use of an online environment avoided certain biases that can occur with human research, specifically the social desirability bias, where participants engage in or report more pro-social behaviour and under report or refrain from anti-social behaviour. Our study thus provides a unique perspective into female-directed hostility.

While playing with male teammates, men generally follow rules associated with navigating hierarchies. Skill did not moderate focal player positivity towards a male-voiced teammate, but higher skilled individuals were less negative. In addition, when performing poorly, players increased the number of positive and neutral statements (Table 1), and were generally less negative towards a male-voiced teammate (Fig 3). As decreased cooperation or behaviours that lead to failure are often punished by teammates [35,36], the increase of positive and neutral statements and relatively less-frequent use of negative statements suggests poor-performing, lower-skilled males are demonstrating submissive behaviour towards a male-voiced teammate.

Males behaved in the opposite manner when playing with a female-voiced teammate. Overall, the female-voiced manipulation experienced a greater frequency of negative comments, but female-directed negativity decreased as focal-player performance improved (Fig 3). In addition, focal-player skill further moderated player behaviour with the lowest-skilled males behaving less positively towards a female voice. Focal players also increased the use of positive statements as their skill increased (Figs 1 & 2). Taken together, these results suggest that it is lower-skilled poorer-performing males that are significantly more hostile towards females, and higher-skilled focal players are more supportive.

Our results support an evolutionary argument for why low-status, low-performing males are hostile towards female competitors. Dominance is tightly linked to fitness in men as studies from hunter-gatherer societies demonstrate that dominance rank increases fitness through offspring number [14] and resource availability [12]. Even in modern day society, dominance and not attractiveness is associated with college male mating success [13]. Low-status and low-performing males have the most to lose as a consequence of the hierarchical reconfiguration due to the entry of a competitive woman. As men often rely on aggression to maintain their dominant social status [37], the increase in hostility towards a woman by lower-status males may be an attempt to disregard a female’s performance and suppress her disturbance on the hierarchy to retain their social rank. This idea is reinforced by the fact that higher-skilled males that should not feel threatened by a female increased their number of positive comments.

Apart from restructuring the hierarchy, a high status female poses a secondary threat to relatively lower status males: as women are attracted to dominance [13], a high-status female is less likely to find lower-status males attractive. We argue that a secondary benefit of increased female-directed hostility is that it simultaneously decreases a female’s confidence and perception of her self-worth (i.e. negging) while simultaneously increasing the perception of him being a dominant (i.e. socially valuable) mate. Higher-skilled (i.e. more dominant) males do not behave in this manner as there is no need for them to reinforce their dominance to maintain their attractiveness. Although there is no direct evidence in the literature that negative behaviour towards females increases a male’s mating opportunity, our results provide an interesting testable hypothesis requiring further investigation.

There are two alternative social explanations for our results. First, players could be responding to the novelty of having a female voiced teammate in a male-dominated environment. Indeed, a female speaking while playing first-person shooter games is rare as none of the 189 players recorded were female. However, novelty is not the driver of the differences in behaviour as performance and skill mediated the number of positive and negative statements. Second, individuals may simply be responding more aggressively towards individuals with a higher pitched voice (a female in this case), as dominance is predictable by voice pitch [38]. Although we cannot rule out this possibility, it’s still in line with our finding that individuals feel more threatened by lower-status individuals.

Our study demonstrates that video games offer a unique opportunity to examine variation in sexist behaviours and the situations that result in changes in sexist attitudes. By demonstrating that female-directed hostility primarily originates from low-status, poorer-performing males, our results suggest that a way to counter it may be through teaching young males that losing to the opposite sex is not socially debilitating. This can be most easily done using video games as the gaming population is nearly equally split between men (52%) and women (48%) [21]. However, given that there is a general bias in game preferences between the sexes [39], first-person shooters largely remain male-biased communities not unlike some male dominated work and study environments (e.g., the technology sector, engineering, the mining industry) where women experience greater frequencies of sexist behaviour. The idea that videogames may be reinforcing such gender segregation as the norm for many teenagers is troubling given the fact that a significant proportion of them are gamers. Such ideas have the potential to spill over in real-life interactions [40] and promote socially unacceptable behaviours such as sexism.

## Supporting Information

S1 Dataset Dataset for analyses. (CSV)

S1 Text Language used in audio recordings. (DOCX)

S2 Text Example comments from other players. (DOCX)
