# Correction: Insights into Sexism: Male Status and Performance Moderates Female-Directed Hostile and Amicable Behaviour

**DOI:** 10.1371/journal.pone.0138399

**Published:** 2015-09-14

**Authors:** Michael M. Kasumovic, Jeffrey H. Kuznekoff

In the Funding section, the funding statement is incorrect. The authors received no specific funding for this work.
