# Supplementary material for: Insights into Sexism: Male Status and Performance Moderates Female-Directed Hostile and Amicable Behaviour
Source: PLoS One. 2015 Jul 15;10(7):e0131613. doi: 10.1371/journal.pone.0131613 (PMC4503401; doi:10.1371/journal.pone.0131613)
Supplement: S1 Text — (DOCX) [file pone.0131613.s002.docx]

**S1 File. Language used in audio recordings**

**Pre-game**

           “Hi everybody”

           “I like this map”

           “Alright team let’s do this”

**In-game**

           “Nice job so far”

           “Nice shot there”

           “That was a great kill you just had”

           “This game is going good so far”

           “I think I just saw a couple of them heading this way”

**Post-game**

“That was a good game everyone”

          “I had fun playing that game”

“Thanks for the game, bye”

**Miscellaneous**

“Yes”

“No”

“I can’t talk right now”
