# Supplementary material for: Insights into Sexism: Male Status and Performance Moderates Female-Directed Hostile and Amicable Behaviour
Source: PLoS One. 2015 Jul 15;10(7):e0131613. doi: 10.1371/journal.pone.0131613 (PMC4503401; doi:10.1371/journal.pone.0131613)
Supplement: S2 Text — (DOCX) [file pone.0131613.s003.docx]

**S2 File. Example comments from other players**

**Negative-Female**

“Lasher dude you suck dick.”

“Yeah, stop stealing my kills, you little piece of shit.”

“Shut up, you whore. She’s a nigger though.”

“Should’ve made me a sandwich, bitch.”

“It’s the bitch stealing my kills.”

**Negative-Male**

“Lasher, you sucked.”

“Freaking retard.”

“I liked your lag trick, jackass.”

“You suck dick.”

“Bitch had my laser, thank you very much.”

**Positive-Female**

“Ooh, you should nice.”

“After this game party up.”

“Do ya thing, girl.”

“Yeah, that was beautiful.”

“I love you, I love you.”

**Positive-Male**

“Party up.”

“Good game, guys.”

“Good game Lasher.”

“I’m so proud of you being the highest level player in here.”

“That was awesome, that was so sick.”

**Neutral-Female**

“Are you there?”

“Lasher man, what’s up?”

“Are you good at this game?”

“Lasher or whatever, what’s your gamertag?”

**Neutral-Male**

“You wanna jump in the jeep?”

“Are you gonna use them rocket?”

“You guys wanna team?”

“From where, which direction?”
